# Role of Preoperative Information and Education of Patients Undergoing Total Hip Arthroplasty: A Narrative Review of the Literature

**DOI:** 10.7759/cureus.66094

**Published:** 2024-08-03

**Authors:** Sara Eleni Amprachim, John Vlamis, Vasileios S Nikolaou, Spyros G Pneumaticos

**Affiliations:** 1 3rd Department of Orthopaedics, National and Kapodistrian University of Athens, KAT Attica General Hospital, Athens, GRC; 2 2nd Department of Orthopaedics, National and Kapodistrian University of Athens School of Medicine, Athens, GRC

**Keywords:** postoperative anxiety, postoperative pain, preoperative information, preoperative education, total hip arthroplasty

## Abstract

Total hip arthroplasty (THA) is a common and highly effective surgical procedure used to relieve pain and improve function in patients with severe hip arthritis and other hip disorders. While the surgical techniques and implants used in THA have advanced significantly, the importance of preoperative information and education cannot be overstated. The aim of this narrative review is to explore the effect of preoperative information and education on the outcome of THA. Key components of preoperative education and information include detailed information about the operation itself, the preoperative preparation, the postoperative pain management and rehabilitation, the possibility of postoperative complications, psychosocial support, and answers to frequently asked questions. The results of the study have confirmed the contradictory findings found in the literature concerning the impact of preoperative education on THA clinical outcomes, including pain, anxiety, functionality, postoperative rehabilitation, duration of hospitalization, and rate of complications. While, theoretically, preoperative education should have a positive effect on clinical outcomes, a plethora of studies have failed to support this hypothesis. Thus, there is a great need for properly designed, prospective, randomized, and controlled studies that have sufficient power in order to fully elucidate the role of preoperative education and information on THA outcomes.

## Introduction and background

Total hip arthroplasty (THA) has been characterized as the operation of the last century [1]. It involves the replacement of a damaged hip joint with prosthetic implants made of metal, ceramics, and polymers, including an acetabular component, a femoral component, and a bearing surface. This procedure is typically recommended for patients who suffer from severe osteoarthritis, rheumatoid arthritis, avascular necrosis, or other debilitating hip conditions that do not respond to conservative treatment [1]. THA goals include pain relief, restoration of function and mobility, correction of deformities, and improvement of quality of life. Despite the increased application of THA and advances in surgical techniques and implants, expectations and satisfaction remain unfulfilled for a large number of patients [2].

Preoperative education is defined as “any educational intervention provided before surgery that aims to improve people's knowledge, behaviors, and health outcomes” [3]. It has been widely studied as an important factor influencing patient expectations of surgery and postoperative recovery. Preoperative patient education and information have been recognized as an essential component of rehabilitation programs. It may be provided by surgeons, nurses, physiotherapists, or psychologists. Education may include analysis of the relevant anatomy, surgical steps, potential risks and complications, hospital discharge planning, expected outcomes, postoperative pain management, and early recovery programs when indicated [3-4]. Preoperatively, psychological preparation and education appear to be useful in reducing hospital length of stay and minimizing medications and stress, as well as depression [5].

Preoperative education has been applied successfully to a wide range of surgeries, including abdominal, orthopedic, and heart surgeries [6]. Reported benefits include a reduction in postoperative pain, stress, and anxiety, functional improvements, rehabilitation enhancement, shorter duration of hospitalization, and lower healthcare costs [7-10]. Recent literature shows that patients who attend pre-surgical education classes are able to reduce their anxiety and have better postoperative pain control, more realistic expectations, and a better understanding of surgery. Educated patients are more likely to follow preoperative and postoperative instructions, leading to faster recovery and fewer complications [3-4,6]. However, the impact of preoperative education on orthopedic patients has not been fully clarified [11]. The aim of this narrative literature review is to summarize the role of preoperative patients’ information and education in the clinical outcome of THA.

## Review

Components of preoperative information and education

Effective preoperative education programs for THA should be comprehensive, patient-centered, and multimodal. Key components include detailed information about the operation itself, the preoperative preparation, the postoperative pain management and rehabilitation, the possibility of postoperative complications, psychosocial support, and answers to frequently asked questions [12]. Preoperative education and information can be provided by surgeons themselves, physiotherapists, nurses, or psychologists [3].

Preoperatively, patients should be informed about preoperative medical assessments, such as blood tests and imaging studies; the initiation or discontinuation of certain drugs, such as anticoagulants; and preoperative lifestyle changes, such as smoking cessation, weight management, and physical conditioning. The patients should be clearly informed about the duration of preoperative fasting. Education on prehabilitation and detailed instructions on specific exercises for the preoperative strengthening of the hip and surrounding muscles are essential [13-14].

Concerning the operation, patients should receive detailed information about hip anatomy and the surgical procedure, including a step-by-step explanation of hip arthroplasty, an analysis of the different types of hip implants, and the rationale for the choice of a specific type. Patients should be informed about the type of anesthesia (general or regional) and what to expect before, during, and after anesthesia [13-14].

The most important element of preoperative information, essential for optimal recovery, is comprehensive education on postoperative care and rehabilitation [15]. Patients should be informed about the possibility of postoperative pain along with pain relief options and the adverse events of necessary analgesic drugs. They should be instructed about the care of the surgical wound, stitch removal, use of assistive devices, the necessity of activity restrictions, the gradual implementation of a rehabilitation regime, and the need for follow-up visits [16]. Special attention should be paid to the awareness of potential complications, such as prevention and identification of wound infection, hip dislocation, and thromboembolic events. Patients should also be instructed on the restrictions on certain movements to prevent dislocation [17-18].

Using multiple methods to deliver education ensures that the information is accessible and comprehensible to all patients. Some studies support a live class with in-person educators to teach the material and more importantly answer any remaining questions [19-20]. Apart from a face-to-face explanation and information, brochures, booklets, and informational handouts along with diagrams, videos, and animations explaining the procedure and recovery process can be used [14,21-22]. Online portals, applications, and telehealth consultations for continuous education and support can be used additionally [23-24]. Finally, educating family members about their role in supporting the patient through the surgical journey is of vital importance. Guidance in preparing the home for postoperative recovery, including setting up a safe environment, arranging for help with daily activities, and ensuring access to necessary equipment is essential for an optimal outcome [25].

Impact of preoperative information and education on the THA outcome

Preoperative information and education play a vital role in preparing patients physically, mentally, and emotionally for surgery and the subsequent recovery period. Most studies have shown that well-informed patients tend to have better surgical outcomes including improved pain management, reduced anxiety, faster recovery, shorter duration of hospitalization, lower complication rates, and increased patient satisfaction. However, there are some studies where the beneficial role of preoperative education has not been proven. It seems that results depend on the study design and the educational regimes.

Pain

THA is a procedure accompanied by moderate-to-severe postoperative pain. Theoretically, educated patients are better prepared to manage postoperative pain through a combination of prescribed medications, physical therapy, and other pain relief techniques. Educating patients about the normal levels of pain to expect after THA can help them mentally prepare, which can mitigate their perception of pain. Moreover, knowing that pain will gradually decrease over time can provide reassurance and reduce anxiety-related pain amplification. However, literature studies show contradictory results.

Three prospective randomized studies found significantly decreased pain intensity in patients having education, before THA [13,26-27]. Pinskiy et al. stated that preoperative physical therapy education resulted in less pain on the second postoperative day [13]. In a small study, Krupic et al. suggested that preoperative education provided sufficient pain relief in THA patients [28]. On the other hand, six studies found no significant decrease in pain intensity [20,22,29-32], but Doering et al. suggested that patients receiving preoperative education were better able to deal with pain with reduced need for painkillers [29]. A retrospective analysis of the Swedish Hip Arthroplasty Register noticed that the impact of patients’ education on one-year postoperative pain is minor [31].

A Cochrane review in 2014 calculated that preoperative education was associated with a mean 0.34 point lower VAS scale and a mean 4.84 points lower Western Ontario and McMaster Universities Arthritis Index (WOMAC) score, without establishing statistical significance [3]. A later meta-analysis, published in 2017, concluded that the impact of preoperative education has a significant impact on pain reduction in THA patients (p = 0.017) [33].

Anxiety

Anxiety and stress before THA can negatively impact the surgical experience and recovery. According to Kearney et al., “anxiety increases sensitivity to pain and reduced anxiety lessens complaints of pain” [34]. Less anxiety does not affect pain intensity significantly but improves patients’ ability to cope with pain [34]. Well-informed patients are less likely to cancel or postpone surgeries at the last minute due to misunderstandings or fear. Preoperative education may help in reducing stress and anxiety by providing clarity, setting realistic expectations, and building trust among the patients and the healthcare providers. Detailed information about the surgical procedure, anesthesia, hospital stay, and recovery process reduces the fear of the unknown. Understanding what to expect preoperatively, intraoperatively, and postoperatively can alleviate fear and anxiety [3].

Four studies reported that preoperative education resulted in significantly less anxiety in patients undergoing THA [13,21,26,29], whereas four studies did not find any significant association with anxiety reduction [20,22,35-36]. A preoperative educational program resulted in lower depressive inclinations in patients undergoing THA [25]. A prospective, randomized trial by Lilja et al. found no difference in postoperative anxiety after preoperative education. However, preoperative information was associated with lower cortisol levels on the day of operation and on the first postoperative day, reflecting lower levels of stress [27]. A Cochrane review in 2014 observed that the overall reduction in preoperative anxiety was statistically significant. The authors emphasized that these results should be treated with caution as the effect was small and the studies were not blind [3]. On the contrary, a systematic review in 2017 calculated that the impact of preoperative education was not statistically significant (p = 0.128) [33]. It seems that the impact of preoperative education on anxiety depends on the type of education technique and the education provider [19-20,26].

Rehabilitation

Knowledge of rehabilitation exercises and postoperative care can accelerate recovery. Patients who understand the importance of early mobilization and adherence to rehabilitation protocols are likely to recover more quickly. Providing a detailed timeline for rehabilitation milestones helps patients set realistic expectations and track their progress, which can encourage adherence to rehab protocols. Preoperative education may prepare THA patients psychologically by providing them with clear expectations of the rehabilitation process [37]. However, McGregor et al. found no significant difference between the education group and the control group in postoperative mobility [20]. Educated patients were found to be able to mobilize and stand sooner after THA, as preoperative education underlined the importance of walking soon after surgery [16,26]. In a prospective study by Vukomanovic et al., pre-educated patients, younger than 70 years, could walk up and down stairs and use the toilet and chair significantly earlier than the control group [30]. Quadriceps strength was significantly higher in pre-educated patients, up to three months postoperatively [33].

Expectations

Unrealistic expectations often cause patient dissatisfaction, creating a gap between patients and surgeons [38]. THA patients tend to have high expectations for the results of the operation [39]. By understanding the potential outcomes, risks, and benefits, patients can set realistic expectations, which can reduce disappointment and anxiety. In patients undergoing THA, positive expectations about postoperative pain and the fulfillment of expectations concerning postoperative functionality are associated with higher global effectiveness ratings [40]. Preoperative education may improve patient preoperative expectations [41]. Three studies found an association between preoperative education and higher levels of satisfaction and more realistic expectations of surgery, but without statistical differences in the quality of life [20-22].

Duration of hospitalization

Informed patients tend to have shorter hospital stays as they are more proactive in their recovery, leading to reduced healthcare costs and better utilization of hospital resources. Educated patients are more likely to adhere to postoperative care guidelines, including wound care, medication schedules, and rehabilitation exercises, which can prevent complications and expedite discharge. Education on recognizing signs of potential complications enables patients to take prompt action, reducing the likelihood of extended hospitalization [14,28,42].

Seven studies confirmed that preoperative patients’ education before THA led to a significant reduction in the duration of hospitalization [14-16,20,28,42-43]. In a study by Yoon et al., preoperative education before THA was associated with a significantly shorter length of stay (mean reduction of 0.8 days, p < 0.001) [43]. Similarly, a retrospective study by Moulton et al. found that patients attending preoperative education were associated with a significantly reduced duration of hospitalization (mean reduction of 0.74 days, p = 0.046) [16]. The corresponding mean difference in the duration of hospitalization was 0.6 days, after a preoperative education regime with printed guides and a video CD [14]. A meta-analysis in 2017 concluded that preoperative education was associated with reduced length of stay (p = 0.027).

On the other hand, seven other studies reported an insignificant reduction in the duration of hospitalization after preoperative education [13,19,21-22,26,29-30]. This lack of association may reflect the fact that preoperative education may not affect the fulfillment of functional discharge criteria. In addition, there are several variables associated with increased duration of hospitalization, such as gender, advanced age, obesity, multiple medical comorbidities, and the interval between surgery and mobilization. Moreover, even in a well-educated patient, early postoperative mobilization may depend mostly on the use of analgesic drugs and physical therapy.

Complications

Preoperative education focusing on recognizing signs of complications, such as infections, dislocations, or thromboembolic events, can lead to earlier interventions and reduced complications. Moreover, pre-educated patients may more efficiently avoid improper limb positions, preventing dislocations. Patients who feel informed and involved in their care may avoid postoperative complications and are generally more satisfied with their surgical outcomes. Informed patients are more likely to adhere to preoperative and postoperative instructions, which is crucial for optimal recovery [44].

Preoperative education has been associated with a threefold lower incidence of dislocation in the first six months after THA [18]. A Cochrane review in 2014 suggested that preoperative information resulted in 10% fewer adverse events [3]. However, two studies found no difference in complication rate among patients who received preoperative education and control group [15,45].

Cost

Patient participation in preoperative education programs can significantly reduce the overall cost of primary THA. Through pre-operative education, patients are mobilized faster and return to their place of residence sooner, while at the same time, the hospital also benefits from reducing the cost of caring for these patients. Educating patients on the recognition of early signs of complications can prevent severe issues that require readmission. Fewer readmissions translate into lower overall healthcare costs. Preoperative education results in a 27-28% reduced cost, in comparison to patients who did not receive preoperative education before THA [46-47]. A study by Moulton et al. reported a cost saving greater than $12,000 per year among patients who received an education course prior to THA [16].

Discussion

The present study aimed to analyze the role of preoperative information and education in THA. The results of the study have confirmed the contradictory findings concerning the impact of preoperative education on THA clinical outcomes, including pain, anxiety, functionality, postoperative rehabilitation, duration of hospitalization, and rate of complications. While, theoretically, preoperative education should have a positive effect on clinical outcomes, a plethora of studies have failed to support this hypothesis.

Clinical studies evaluating the effect of preoperative education on THA clinical outcomes have several design limitations. In comparative studies, patients from the control groups cannot be prevented from seeking information on their own. These control groups may have access to the same educational data as the intervention groups - through friends, family, the Internet, and previous experiences. Moreover, for ethical reasons, patients in the control groups cannot be denied answering questions posed by nurses and medical staff. All these methodological flaws contribute to limitations that weaken the quality of the studies.

Drawn conclusions from the present review may be flawed by the general heterogeneity of the pooled studies. There is a great heterogeneity concerning the methods of preoperative education, which cannot be standardized. Descriptions of preoperative education programs for THA are limited, and effective program design is currently unknown. There are limited studies that only examine preoperative patient education and its role. Thus, there is a great need for properly designed, prospective, randomized, controlled studies with sufficient power to be able to generalize to the wider population.

As preoperative education can influence postoperative patient outcomes, it is important to formulate a well-designed preoperative program that is individualized and takes into account patient perceptions. Many times, patients’ perceptions during the procedure are different from the expectations of the healthcare professionals. For this reason, healthcare professionals have an important role in shaping a positive experience for patients. Therefore, the need for preoperative education before the admission of patients to the hospital is imperative, which should start as soon as the initial decision to perform the surgery is made. In our opinion, it should continue during the preoperative checkup and be completed on the day of surgery.

## Conclusions

THA is performed in vast numbers globally, and many efforts have been made for the improvement of clinical outcomes. Preoperative information and education may play a critical role in the success of THA. Patients’ provision with comprehensive, personalized, and accessible education can enhance patient outcomes, reduce anxiety, and improve overall satisfaction. However, literature results draw contradictory conclusions. Effective preoperative education programs require a multidisciplinary approach, utilization of technology, and continuous evaluation and improvement. As the field of preoperative education continues to evolve, innovations such as virtual reality, telehealth, personalized medicine, and artificial intelligence hold promise for further enhancing the patient experience and outcomes in THA.

## References

[1] Learmonth ID, Young C, Rorabeck C (2007). The operation of the century: total hip replacement. Lancet.

[2] Scott CE, Clement ND, Davis ET, Haddad FS (2022). Modern total hip arthroplasty: peak of perfection or room for improvement?. Bone Joint J.

[3] McDonald S, Page MJ, Beringer K, Wasiak J, Sprowson A (2014). Preoperative education for hip or knee replacement. Cochrane Database Syst Rev.

[4] McDonald S, Hetrick S, Green S (2004). Pre-operative education for hip or knee replacement. Cochrane Database Syst Rev.

[5] Fortner PA (1998). Preoperative patient preparation: psychological and educational aspects. Semin Perioper Nurs.

[6] Darville-Beneby R, Lomanowska AM, Yu HC (2023). The impact of preoperative patient education on postoperative pain, opioid use, and psychological outcomes: a narrative review. Can J Pain.

[7] Kruzik N (2009). Benefits of preoperative education for adult elective surgery patients. AORN J.

[8] Oshodi TO (2007). The impact of preoperative education on postoperative pain. Part 2. Br J Nurs.

[9] Oshodi TO (2007). The impact of preoperative education on postoperative pain. Part 1. Br J Nurs.

[10] Walker JA (2007). What is the effect of preoperative information on patient satisfaction?. Br J Nurs.

[11] Johansson K, Nuutila L, Virtanen H, Katajisto J, Salanterä S (2005). Preoperative education for orthopaedic patients: systematic review. J Adv Nurs.

[12] Edwards PK, Mears SC, Lowry Barnes C (2017). Preoperative education for hip and knee replacement: never stop learning. Curr Rev Musculoskelet Med.

[13] Pinskiy M, Lubovsky O, Kalichman L (2021). The effect of a preoperative physical therapy education program on short-term outcomes of patients undergoing elective total hip arthroplasty: a controlled prospective clinical trial. Acta Orthop Traumatol Turc.

[14] Yeh ML, Chen HH, Liu PH (2005). Effects of multimedia with printed nursing guide in education on self-efficacy and functional activity and hospitalization in patients with hip replacement. Patient Educ Couns.

[15] Siggeirsdottir K, Olafsson O, Jonsson H, Iwarsson S, Gudnason V, Jonsson BY (2005). Short hospital stay augmented with education and home-based rehabilitation improves function and quality of life after hip replacement: randomized study of 50 patients with 6 months of follow-up. Acta Orthop.

[16] Moulton LS, Evans PA, Starks I, Smith T (2015). Pre-operative education prior to elective hip arthroplasty surgery improves postoperative outcome. Int Orthop.

[17] Brady OH, Masri BA, Garbuz DS, Duncan CP (2000). Rheumatology: 10. Joint replacement of the hip and knee--when to refer and what to expect. CMAJ.

[18] Lübbeke A, Suvà D, Perneger T, Hoffmeyer P (2009). Influence of preoperative patient education on the risk of dislocation after primary total hip arthroplasty. Arthritis Rheum.

[19] Johansson K, Salanterä S, Katajisto J (2007). Empowering orthopaedic patients through preadmission education: results from a clinical study. Patient Educ Couns.

[20] McGregor AH, Rylands H, Owen A, Doré CJ, Hughes SP (2004). Does preoperative hip rehabilitation advice improve recovery and patient satisfaction?. J Arthroplasty.

[21] Butler GS, Hurley CA, Buchanan KL, Smith-VanHorne J (1996). Prehospital education: effectiveness with total hip replacement surgery patients. Patient Educ Couns.

[22] Clode-Baker E, Draper E, Raymond N, Haslam C, Gregg P (1997). Preparing patients for total hip replacement: a randomized controlled trial of a preoperative educational intervention. J Health Psychol.

[23] Dayucos A, French LA, Kelemen A, Liang Y, Sik Lanyi C (2019). Creation and evaluation of a preoperative education website for hip and knee replacement patients-a pilot study. Medicina (Kaunas).

[24] Bahadori S, Wainwright TW, Ahmed OH (2020). Smartphone apps for total hip replacement and total knee replacement surgery patients: a systematic review. Disabil Rehabil.

[25] Huang TT, Sung CC, Wang WS, Wang BH (2017). The effects of the empowerment education program in older adults with total hip replacement surgery. J Adv Nurs.

[26] Giraudet-Le Quintrec JS, Coste J, Vastel L (2003). Positive effect of patient education for hip surgery: a randomized trial. Clin Orthop Relat Res.

[27] Lilja Y, Rydén S, Fridlund B (1998). Effects of extended preoperative information on perioperative stress: an anaesthetic nurse intervention for patients with breast cancer and total hip replacement. Intensive Crit Care Nurs.

[28] Krupic F, Määttä S, Garellick G, Lyckhage ED, Kärrholm J (2012). Preoperative information provided to Swedish and immigrant patients before total hip replacement. Med Arch.

[29] Doering S, Katzlberger F, Rumpold G (2000). Videotape preparation of patients before hip replacement surgery reduces stress. Psychosom Med.

[30] Vukomanović A, Popović Z, Durović A, Krstić L (2008). The effects of short-term preoperative physical therapy and education on early functional recovery of patients younger than 70 undergoing total hip arthroplasty. Vojnosanit Pregl.

[31] Torisho C, Mohaddes M, Gustafsson K, Rolfson O (2019). Minor influence of patient education and physiotherapy interventions before total hip replacement on patient-reported outcomes: an observational study of 30,756 patients in the Swedish Hip Arthroplasty Register. Acta Orthop.

[32] Gocen Z, Sen A, Unver B, Karatosun V, Gunal I (2004). The effect of preoperative physiotherapy and education on the outcome of total hip replacement: a prospective randomized controlled trial. Clin Rehabil.

[33] Moyer R, Ikert K, Long K, Marsh J (2017). The value of preoperative exercise and education for patients undergoing total hip and knee arthroplasty: a systematic review and meta-analysis. JBJS Rev.

[34] Kearney M, Jennrich MK, Lyons S, Robinson R, Berger B (2011). Effects of preoperative education on patient outcomes after joint replacement surgery. Orthop Nurs.

[35] Sjöling M, Nordahl G, Olofsson N, Asplund K (2003). The impact of preoperative information on state anxiety, postoperative pain and satisfaction with pain management. Patient Educ Couns.

[36] Daltroy LH, Morlino CI, Eaton HM, Poss R, Liang MH (1998). Preoperative education for total hip and knee replacement patients. Arthritis Care Res.

[37] Loft M, McWilliam C, Ward-Griffin C (2003). Patient empowerment after total hip and knee replacement. Orthop Nurs.

[38] Colibazzi V, Coladonato A, Zanazzo M, Romanini E (2020). Evidence based rehabilitation after hip arthroplasty. Hip Int.

[39] Mahomed NN, Liang MH, Cook EF, Daltroy LH, Fortin PR, Fossel AH, Katz JN (2002). The importance of patient expectations in predicting functional outcomes after total joint arthroplasty. J Rheumatol.

[40] Kästner A, Ng Kuet Leong VS, Petzke F, Budde S, Przemeck M, Müller M, Erlenwein J (2021). The virtue of optimistic realism - expectation fulfillment predicts patient-rated global effectiveness of total hip arthroplasty. BMC Musculoskelet Disord.

[41] Mancuso CA, Graziano S, Briskie LM, Peterson MG, Pellicci PM, Salvati EA, Sculco TP (2008). Randomized trials to modify patients' preoperative expectations of hip and knee arthroplasties. Clin Orthop Relat Res.

[42] Crowe J, Henderson J (2003). Pre-arthroplasty rehabilitation is effective in reducing hospital stay. Can J Occup Ther.

[43] Yoon RS, Nellans KW, Geller JA, Kim AD, Jacobs MR, Macaulay W (2010). Patient education before hip or knee arthroplasty lowers length of stay. J Arthroplasty.

[44] Klaiber U, Stephan-Paulsen LM, Bruckner T (2018). Impact of preoperative patient education on the prevention of postoperative complications after major visceral surgery: the cluster randomized controlled PEDUCAT trial. Trials.

[45] Santavirta N, Lillqvist G, Sarvimäki A, Honkanen V, Konttinen YT, Santavirta S (1994). Teaching of patients undergoing total hip replacement surgery. Int J Nurs Stud.

[46] Jones S, Alnaib M, Kokkinakis M, Wilkinson M, St Clair Gibson A, Kader D (2011). Pre-operative patient education reduces length of stay after knee joint arthroplasty. Ann R Coll Surg Engl.

[47] Sigurdsson E, Siggeirsdottir K, Jonsson H Jr, Gudnason V, Matthiasson T, Jonsson BY (2008). Early discharge and home intervention reduces unit costs after total hip replacement: results of a cost analysis in a randomized study. Int J Health Care Finance Econ.

